# *Interleukin-4* Gene Polymorphisms in Romanian Patients with Inflammatory Bowel Diseases: Association with Disease Risk and Clinical Features

**DOI:** 10.3390/diagnostics13081465

**Published:** 2023-04-18

**Authors:** Andrei Ovidiu Olteanu, Elena Mirela Ionescu, Cristian George Tieranu, Luis Ovidiu Popa, Silvia Ioana Andrei, Carmen Monica Preda, Monica Irina Dutescu, Mihai Bojinca, Ioana Tieranu, Olivia Mihaela Popa

**Affiliations:** 1 Department of Gastroenterology, “Carol Davila” University of Medicine and Pharmacy, 020021 Bucharest, Romania; ovidiu-andrei.olteanu@drd.umfcd.ro (A.O.O.); mirela.ionescu@umfcd.ro (E.M.I.); carmen.preda@umfcd.ro (C.M.P.); 2 Department of Gastroenterology, “Elias” Emergency University Hospital, 011461 Bucharest, Romania; 3 Molecular Biology Department, “Grigore Antipa” National Museum of Natural History, 011341 Bucharest, Romania; popaluis@antipa.ro; 4 Clinic of Internal Medicine II, Thüringen-Kliniken “Georgius Agricola”, 07318 Saalfeld, Germany; andrei_silvia_ioana@yahoo.com; 5 “Prof. Dr. C. T. Nicolau” National Institute of Blood Transfusion, 011155 Bucharest, Romania; monica_dutescu@yahoo.com; 6 Department of Rheumatology and Internal Medicine, “Carol Davila” University of Medicine and Pharmacy, 020021 Bucharest, Romania; mihai.bojinca@umfcd.ro; 7 Department of Pediatrics, “Carol Davila” University of Medicine and Pharmacy, 020021 Bucharest, Romania; dr.cindeaioana@yahoo.ro; 8 Department of Immunology and Pathophysiology, “Carol Davila” University of Medicine and Pharmacy, 020021 Bucharest, Romania; olivia.popa@umfcd.ro

**Keywords:** inflammatory bowel disease, crohn’s disease, ulcerative colitis, single nucleotide polymorphism, interleukin-4

## Abstract

1. Introduction. Multiple cytokines have been studied for their role in the propagation of the inflammatory process related to inflammatory bowel diseases (IBD), but the role of interleukin-4 remains controversial. The aim of this study was to evaluate the role of two *IL-4* gene single nucleotide polymorphisms (SNPs) in disease susceptibility and phenotypic expression. 2. Materials and Methods. A group of 160 patients with IBD (86CD/74UC) and 160 healthy controls were genotyped for *IL-4* rs2243250/−590C/T and rs2070874/−34C/T using real-time polymerase chain reaction with TaqMan assay. 3. Results. The analysis of IBD patients and controls revealed a significantly reduced frequency of the minor allele T of both SNPs in CD patients (*p* = 0.03, OR 0.55 and *p* = 0.02, OR 0.52) and for the entire IBD group (*p* = 0.01, OR 0.57 and *p* = 0.01, OR 0.55). Haplotype analysis identified the most frequent haplotype (rs2243250/rs2070874 CC) associated with a high risk for developing IBD (either UC or CD) (*p* = 0.003). IBD patients with extraintestinal manifestations had significantly increased frequency of the minor alleles T. We also found an association between the presence of allele C of rs2070874 and response to antiTNF treatment. 4. Conclusions. This is the first study to investigate the *IL-4* gene’s relation to IBD susceptibility conducted in Romania. Both SNPs were found to be associated with disease susceptibility and phenotypic features, such as extraintestinal manifestations and response to antiTNF agents.

## 1. Introduction

Inflammatory bowel diseases (IBD) are chronic inflammatory gastrointestinal disorders of unknown etiology that predominantly affect the young population. They are mainly represented by ulcerative colitis (UC) and Crohn’s disease (CD) [1]. IBD patients are prone to developing extraintestinal manifestations (EIM) consisting of musculoskeletal, cutaneous, ocular, and hepatobiliary conditions [2]. Despite extensive research on therapeutic agents for IBD treatment, a significant percentage of patients fail to achieve clinical remission. Inflammatory bowel diseases have become a global disease in the 21st century, with the highest reported prevalence values in Europe and North America [3].

The hypothesis regarding the disease’s pathogenic mechanism implies exposure to environmental factors and dysbiosis as determinants of abnormal immune response in a genetically predisposed host [4]. Patients carrying predisposition alleles to IBD manifest a disproportionate immune response to intestinal microbiota, thus developing IBD symptoms [5].

The role of T lymphocytes in immune response regulation is well established, with a crucial role for T helper 1 (Th1) and 2 (Th2) imbalances in the initiation and progression of bowel inflammation once the intestinal epithelium is injured by external factors [6,7]. Th1-activated lymphocytes promote intestinal inflammation through the production of cytokines with proinflammatory activity, such as IFN-gamma and interleukin-2 (IL-2). The role of Th2 lymphocytes resides in the downregulation of the Th1 activation process through the secretion of IL-4, IL-10, and IL-13 cytokines [8]. Experimental evidence shows that Th2 cells are more abundant in lamina propria of UC patients compared to CD patients, and also in CD patients who do not respond to antiTNF therapy, suggesting that increased Th2 is associated with IBD severity [9,10].

IL-4 plays a key role in promoting T cell growth and differentiation of Th2 cells from naive CD4+ T cells. As a result, levels of IL-4 itself exert an important influence on the balance of cytokines [11]. IL-4 acts as an anti-inflammatory and immunomodulatory factor, especially through its suppression effect over the secretion of proinflammatory cytokines IL-1, IL-6, IL-12, and TNF-alpha [12]. Studies have shown that levels of IL-4 and IL-4 mRNA are reduced in IBD patients, mainly Crohn’s disease patients, indicating a role for this cytokine in the pathophysiological process [13]. IL-4 promotes the healing processes accomplished by macrophages and has an alleviating effect upon patients with colitis, in whom it also limits the activation of monocytes and macrophages in order to prevent the secretion of proinflammatory cytokines, such as IL-1β and TNF-α [14]. Moreover, it has been observed that the administration of an antagonist of IL-4 receptor α for the treatment of atopic dermatitis has caused enteritis as a side effect. These findings lead to the assumption that the immune response mediated by Th2 cells could be protective against IBD [15].

As part of the Th2 locus on chromosome 5q23-31, the *IL-4* gene contains several single nucleotide polymorphisms (SNPs) that have been shown to be associated with increased *IL-4* expression or interference with the splicing sites, thus affecting the protein structure [16,17]. One of these SNPs is −590C/T (rs2243250), a functional polymorphism located in the promoter of the *IL-4* gene. The presence of the T allele introduces a supplementary binding site for NFAT (nuclear factor for activated T cells), which determines a three-fold increase in the level of transcription [11].

*IL-4* SNPs have been extensively investigated in relation to allergic responses, rheumatic disorders, and cancer, but limited research has been conducted in inflammatory bowel disease patients [18,19,20].

This study aimed to investigate the association between *IL-4* gene polymorphisms and susceptibility to IBD in a group of Romanian patients. We also verified the correlations with phenotypic features of the disease.

## 2. Materials and Methods

### 2.1. Study Population and Ethics

This study included a total number of 160 IBD patients (male/female 90/70) consecutively recruited in two referral centers: “Elias“ Emergency University Hospital and Fundeni Clinical Institute, Bucharest, Romania.

Diagnosis of IBD, CD, and UC, respectively, was established based on current accepted guidelines [21,22]. Of the 160 IBD patients included in this study, 86 were CD patients (male/female ratio of 1.15) and 74 were UC patients (male/female ratio of 1.46). The Montreal classification was used for both UC and CD [23]. The following clinical features were recorded: gender, age, disease localization for CD (ileal, ileocolonic, colonic, and upper GI tract), disease behavior for CD (inflammatory, stricturing, and penetrating), extent of UC (proctitis—E1, left-side colitis—E2, and pancolitis—E3), extraintestinal manifestations (presence or absence), need for colectomy in UC, and response to antiTNF-alpha agents. Response to antiTNF treatment was defined as drug persistence at the moment of enrollment. All patients treated with antiTNF had at least 3 months of ongoing treatment. Lack of response was defined as the need to change treatment at 3 months after initiation (primary non-responders). 

The control group consisted of 160 healthy, unrelated subjects (104 M/56F) recruited from the “Prof. Dr. C. T. Nicolau” National Institute of Blood Transfusion, Bucharest, Romania. Control subjects were potential organ donors and had no history of inflammatory bowel disease or a diagnosed immune-related condition based on an in-person questionnaire filled out by each participant prior to enrollment. 

All subjects were unrelated Caucasian individuals of Romanian origin, and written informed consent was obtained prior to enrollment. 

### 2.2. SNP Selection and Genotyping

Two single nucleotide polymorphisms of the *IL-4* gene (rs2243250 and rs2070874) were selected based on an analysis of the available literature as well as by searching the results from the NCBI Database of Short Genetic Variations (dbSNP). The minor allele frequencies (MAF) of the selected SNPs were higher than 10% for samples of European descent.

Blood samples collected on EDTA were used for genomic DNA extraction and purification with QIAamp DNA Blood Mini Kit (Qiagen, Hilden, Germany) following the manufacturer’s protocol.

In total, 320 subjects were screened for *IL-4* gene SNPs by real-time polymerase chain reaction (PCR) with TaqMan Allelic Discrimination Assays (C_16176215_10, C_16176216_10, 7300 Real-Time PCR System, Applied Biosystems by Thermo Fisher Scientific, Foster City, CA, USA) in accordance with the manufacturer’s instructions.

### 2.3. Statistical Analysis

Mann–Whitney U tests were used to compare the variables with non-parametric distribution between groups. For categorical variables, the chi-squared test was applied. Statistical analysis was performed using the Statistical Package for Social Science (SPSS v29.0, IBM Corp., Armonk, NY, USA).

The chi-squared test was used for Hardy–Weinberg equilibrium (HWE) evaluation. Single-marker allelic tests were performed with PLINK v1.07 (https://zzz.bwh.harvard.edu/plink/download.shtml) and the DeFinetti program (http://ihg2.helmholtzmuenchen.de/cgi-bin/hw/hwa1.pl) and *p* values ≤ 0.05 were considered significant. For multiple comparisons, Bonferroni correction was applied [24,25]. 

Haplotypes were estimated using the hap-freq function in PLINK v1.07. The *χ*2 test was used to determine statistical significance. Linkage disequilibrium (LD) between *IL-4* SNPs was evaluated based on genotype data from controls by calculating r^2^ with PLINK v1.07 software.

## 3. Results

A total of 160 patients diagnosed with IBD and 160 sex-matched healthy controls were enrolled (Table 1). Demographic and phenotypical characteristics of patients enrolled in this study are presented in Table 2. The genotyping success rate was 100%. The distribution of the genotypes for the studied SNPs conformed to the Hardy–Weinberg equilibrium (HWE) in both patient and control groups (*p* > 0.05).

We first studied the CD group of patients, where the minor alleles of rs2243250 (T) and rs2070874 (T) were both detected significantly more frequently in the control group compared to the patients (*p* = 0.03, p_corr_ > 0.05 and *p* = 0.02, p_corr_ = 0.04, respectively; Table 3). Moreover, the protective effect of the minor alleles was emphasized in the subgroup of carriers of allele T (CT + TT), who had a lower risk for CD compared to controls (*p* = 0.04 for both studied SNPs).

For the UC group, the frequencies of alleles followed the same pattern as in the CD group, but without reaching the threshold for statistical significance (Table 3). The carriers of the minor allele for both the investigated SNPs showed a significantly lower risk for developing the disease compared to random controls (*p* = 0.04 for rs2243250 and *p* = 0.03 for rs2070874).

The analysis of the whole group of patients with IBD compared to the control group showed a significantly reduced frequency of the minor allele T of rs2243250 in patients (p_corr_ = 0.02). The minor allele T of the second investigated SNP (rs2070874) showed the same protective influence against IBD, as it was significantly over-represented in the healthy controls compared to the patients (p_corr_ = 0.02). Further analysis of the genotypes containing the minor alleles (CT+TT) of both SNPs highlighted the significantly lower risk among carriers of the T allele of developing IBD (*p* = 0.01 for both rs2243250 and rs2070874; Table 3).

Haplotype analysis revealed that only two haplotype combinations had frequencies over 5% in the studied groups (Table 4). The most frequent haplotype (rs2243250/rs2070874 CC) was associated with a high risk of developing IBD (either UC or CD) (*p* = 0.003). The LD between the two investigated SNPs was moderate (r^2^ = 0.72 for the control group).

We further analyzed whether the two polymorphisms at the *IL-4* gene were associated with clinical features of IBD. Disease location, extraintestinal symptoms, and response to biologic therapy were considered for this analysis. 

In the subgroup of CD patients with ileal location (N = 35), the minor allele T for both SNPs had a lower frequency (8.5%) but did not reach a significant value (Table 5). For UC patients with pancolitis (N = 28), the genotype distribution for both SNPs revealed significant differences versus controls. For rs2243250, the homozygote genotypes were over-represented in patients compared with controls (7% versus 1.8% for TT, *p* = 0.008, OR 0.09, 95%CI 0.01–0.76, and 82% versus 67%, *p* = 0.03, OR 0.27, and 95%CI 0.08–0.97). 

Data for extraintestinal manifestations (EIM) were available for 158 patients, of whom 36 (22.7%) had EIM. We observed that minor alleles (T) were predisposed to concurrent extraintestinal symptoms as their frequency for both SNPs was significantly higher in patients with EIM (Table 6). IBD patients with rs2070874 CT and TT genotypes had a particularly increased risk of developing EIM (*p* = 0.004). 

From the IBD study group, 92 patients received antiTNF treatment (64 CD and 28 UC; Table 2). All patients who responded to therapy (N = 65) had at least one copy of the major allele C of rs2070874 compared with only 92.5% of patients without response (*p* = 0.02, OR 0.078, 95%CI 0.004–1.678).

## 4. Discussion

While Th1 and Th17 immune responses have been extensively studied in IBD, the role of Th2 immunity is less investigated and understood. Studies have shown that the type 2 cytokines IL-4 and IL-13 play a major role in macrophage-mediated epithelial wound healing and that they promote colitis alleviation [26,27,28,29]. An analysis of gene expression patterns in the peripheral blood of IBD patients (both CD and UC) revealed that *IL-4* expression was downregulated compared with healthy controls [30]. The level of *IL-4* mRNA was found to be significantly higher in early ileal lesions compared with normal tissues of CD patients or controls, but not in chronic ileal lesions, which suggests different IL-4 contribution depending on the stage of the disease [31]. 

Linkage and genome-wide studies have pointed to several genetic regions as possible factors contributing to IBD susceptibility. The Th2 locus from the 5q23-31 region is of particular interest for this study as this locus has been linked especially to CD [32,33]. Polymorphisms in the promoter region of the *IL-4* gene influence the level of *IL-4* expression, predisposing carriers to an increased severity of IL-4-mediated diseases, but extensive research is necessary to establish the genetic contribution of this cytokine to UC and/or CD. As previously mentioned, the IL-4 pathway contributes to multiple functions, such as cytokine production, inflammation, and tissue adhesion [34].

In this study, we genotyped IBD patients and healthy controls for two *IL-4* gene polymorphisms: *IL-4* rs2243250 (−590C/T) and *IL-4* rs2070874 (−34C/T). Both SNPs were found to confer a protective effect against both CD and UC. The haplotype of the major alleles, rs2243250/rs2070874 CC, was associated with a high risk of developing IBD (either UC or CD).

The available data regarding the role of the *IL-4* gene in IBD susceptibility are scarce. There have been several studies in various populations with inconsistent results, probably due to genetic variability and different disease characteristics [35,36,37,38,39].

For rs2070874 (also known as −34C/T or +33C/T depending on the counting mode), previous data from British populations have revealed contradictory results. While Aithal et al., 2001 found a higher risk for CD conferred by the presence of the minor T allele (*p* = 0.002), Onnie et al., 2006 did not find any association with the risk of CD. We should mention that the subjects for the second British study were 94% white Caucasians [40,41]. Both studies contradict our results. Furthermore, two studies on Caucasian populations from Portugal and New Zealand did not find any significant correlations between *IL-4* gene SNP rs2070874 and CD regarding disease risk, disease location, or the presence of EIM, which also contradicts our data. However, the frequency of the genotypes followed the same pattern as in our population, with CD patients showing lower CT and TT frequency than controls [35,36]. 

The Iranian study by Daryani et al. analyzed three polymorphisms of the *IL-4* gene in IBD patients (−590C/T, −34C/T, and −1098G/T) and reported significant results for the genotype distribution for all SNPs. For −590C/T and −34C/T, the carriers of the T alleles (CT and TT genotypes) were less frequent in IBD patients (22% and 39%) than in controls (92.8% and 56.1%, respectively), similar to our results [42]. 

Consistent with our findings, Klein et al. also reported the association of rs2243250 (−590C/T) with protection against CD in a German population, but not with UC. The frequencies of minor allele carriers (CT and TT) were similar to our groups (30% in controls and 21% in CD patients) [43].

Furthermore, regarding the rs2243250 SNP, previously published data from New Zealand found no association with IBD disease susceptibility [37]. For the same SNP, Connely et al. reported an association with *Clostridium difficile* infection in American IBD patients [38].

Apart from single nucleotide polymorphisms analyses, a study of a Chinese population reported the association of *IL-4* gene 70pb VNTR polymorphism from intron 3 with the risk of UC [39].

We found associations between the two studied *IL-4* SNPs and disease phenotype. We obtained a strong significant correlation between the minor T allele of rs2070874 and the presence of EIM and the lack of response to antiTNF-alpha agents among IBD patients. We also found that TT and CC genotypes of rs2243250 were risk factors for the pancolitis pattern of UC.

To our knowledge, this is the first study reporting a relationship between *IL-4* SNPs and the presence of EIM. Previously published data on phenotypic characteristics influenced by *IL-4* SNPs have found associations only with disease location, familial recurrence, or response to treatment in CD [36,37,42].

Regarding the level of linkage disequilibrium at the investigated locus, the results of our study show a medium LD (r^2^ = 0.72) between the two SNPs. Various reports show complete or strong LD for these polymorphisms (r^2^ > 0.89), but also limited LD (r^2^ = 0.5) depending on the ethnicity [40,44,45].

Taken together, the data reported so far point to a decreased frequency of minor alleles of −590C/T and −34C/T polymorphisms in IBD patients compared to controls, except for in the British population. This means a reduced level of *IL-4* expression generated by the 590*C allele and by other regulatory polymorphisms in linkage disequilibrium, but with a variability attributable to the genetic background of each population. More studies on different ethnic groups are necessary to clarify the implications of IL-4 in IBD etiopathogenesis. 

An important limitation of this study is the low number of patients enrolled. Another limitation may be attributed to the definition used for a lack of response to antiTNF agents because, per our definition, only primary non-responders were included in this group. Our results need confirmation in larger cohorts of patients with IBD.

## 5. Conclusions

This study is the first to investigate the role of *IL-4* gene polymorphisms in IBD in the Romanian population. We found significant associations between the investigated SNPs and disease susceptibility and phenotypic features, such as concomitant EIM. The investigated SNPs also seem to influence the response to antiTNF agents in the studied IBD population. Larger cohort studies are needed to confirm our findings. 

## Figures and Tables

**Table 1:** Baseline characteristics of the IBD and control groups.

	IBD Group (*n* = 160)	Control Group (*n* = 160)	*p*-Value
Gender (%)			
Male	56.3%	65.0%	*p* = 0.10
Female	43.7%	35.0%	
Age (Mean ± SD)	32.29 ± 11.85	37.73 ± 13.75	*p* < 0.001

**Table 2:** Demographic and phenotypic characteristics of enrolled patients.

	Crohn’s Disease (CD)	Ulcerative Colitis (UC)
Patients N (%)	86 (53.75%)	74 (46.25%)
Gender (%) Male Female	53.5% 46.5%	59.5% 40.5%
Age (Mean ± SD)	36 ± 9.60	34.50 ± 13.72
Disease extension (Montreal classification)	L1: 35	E1: 6
	L2: 20	E2: 40
	L3: 30	E3: 28
Disease behavior (for CD)	B1: 39	N/A
	B2: 28	N/A
	B3: 19	N/A
AntiTNF treatment (Y/N)	64/22	28/46
Response to antiTNF treatment (Y/N)	47/17	18/10
Adverse effects of antiTNF treatment N (%)	10 (15.6%)	1 (3.57%)
Presence of extraintestinal manifestations N (%) *	21 (24.7%)	15 (20.27%)
Colectomy (for UC) N (%)	N/A	5 (6.75%)

* Data available for 158 IBD patients.

**Table 3:** Results of the association study of *IL-4* single nucleotide polymorphisms and IBD groups.

SNP	Controls (N = 160)	CD (N = 86)	Statistics	UC (N = 74)	Statistics	IBD (N = 160)	Statistics
rs2243250 Minor allele							
T	56 (17.5%)	18 (10.4%)	OR = 0.55 95%CI 0.31–0.97 ***p* = 0.03**	17 (11.5%)	OR = 0.61 95%CI 0.34–1.09 *p* = 0.09	35 (11%)	OR = 0.57 95%CI 0.36–0.91 ***p* = 0.01**
Genotype							
CT + TT	50 + 3 (33%)	18 + 0 (21%)	OR = 0.53 95%CI 0.28–0.98 ***p* = 0.04**	13 + 2 (20.3%)	OR = 0.51	31 + 2 (20.6%)	OR = 0.52 95%CI 0.31–0.86 ***p* = 0.01**
CC	107 (67%)	68 (79%)		59 (79.7%)	95%CI 0.26–0.98 ***p* = 0.04**	127 (79.4%)	
rs2070874 Minor allele							
T	55 (17.2%)	17 (9.8%)	OR = 0.52 95%CI 0.29–0.94 ***p* = 0.02**	16 (10.8%)	OR = 0.58 95%CI 0.32–1.55 *p* = 0.07	33 (10.3%)	OR = 0.55 95%CI 0.34–0.88 ***p* = 0.01**
Genotype							
CT + TT	47 + 4 (32%)	17 + 0 (19.8%)	OR = 0.52 95%CI 0.28–0.98 ***p* = 0.04**	12 + 2 (19%)	OR = 0.49 95%CI 0.25–0.97	29 + 2 (19.4%)	OR = 0.51 95%CI 0.30–0.85 ***p* = 0.01**
CC	109 (68%)	69 (80.2%)		60 (81%)	***p* = 0.03**	129 (80.6%)	

OR, odds ratio; CI, 95% confidence interval; *p* values < 0.05 are indicated in bold.

**Table 4:** Results of *IL-4* rs2243250/rs2070874 haplotype analyses for the investigated diseased groups.

Haplotype rs2243250/rs2070874	Controls (N = 160)	UC (N = 74)	*p*	CD (N = 86)	*p*	IBD (N = 160)	*p*
CC	80.94%	88.51%	**0.04**	89.53%	**0.01**	89.06%	**0.003**
TT	15.62%	10.81%	0.16	9.8%	0.07	10.31%	**0.04**

Haplotype association tests were performed with PLINK 1.07 software; *p* values < 0.05 are indicated in bold.

**Table 5:** Results for allele frequency differences in IBD patients versus healthy controls depending on disease location.

	IL-4 rs2243250			IL-4 rs2070874		
	*p*	OR	95%CI	*p*	OR	95%CI
CD location						
Ileal	0.06	0.44	0.18–1.07	0.07	0.45	0.18–1.09
Colonic	0.2	0.52	0.17–1.53	0.2	0.53	0.18–1.56
Ileo-colonic	0.4	0.72	0.32–1.61	0.2	0.63	0.27–1.47
UC location						
E1	0.7	0.42	0.05–3.38	0.2	0.19	0.01–3.28
E2	0.1	0.59	0.28–1.26	0.1	0.61	0.28–1.29
E3	0.3	0.67	0.29–1.56	0.3	0.68	0.29–1.60

OR, odds ratio; CI, 95% confidence interval.

**Table 6:** Results of the association study of *IL-4* single nucleotide polymorphisms and EIM in IBD patients.

SNP	Patients without EIM (N = 122)	Patients with EIM (N = 36)	Statistics
rs2243250			
Minor allele			
T	22 (9%)	13 (18.05 %)	OR = 2.22 95%CI 1.05–4.67 ***p* = 0.03**
Genotype			
CT + TT	18 + 2 (16.4%)	13 + 0 (36%)	OR = 2.88 95%CI 1.25–6.62 ***p* = 0.01**
CC	102 (83.6%)	23 (64%)	
rs2070874			
Minor allele			
T	20 (8.2 %)	13 (18.05 %)	OR = 2.46 95%CI 1.16–5.25 ***p* = 0.01**
Genotype			
CT + TT	16 + 2 (14.75%)	13 + 0 (36%)	OR = 3.26 95%CI 1.40–7.59 ***p* = 0.004**
CC	104 (85.25%)	23 (64%)	

OR, odds ratio; CI, 95% confidence interval; *p* values < 0.05 are indicated in bold.

## Data Availability

The raw data supporting the results are available from the corresponding author upon reasonable request.
