# Correction: Olteanu et al. *Interleukin-4* Gene Polymorphisms in Romanian Patients with Inflammatory Bowel Diseases: Association with Disease Risk and Clinical Features. *Diagnostics* 2023, *13,* 1465

**DOI:** 10.3390/diagnostics15050546

**Published:** 2025-02-24

**Authors:** Andrei Ovidiu Olteanu, Elena Mirela Ionescu, Cristian George Tieranu, Luis Ovidiu Popa, Silvia Ioana Andrei, Carmen Monica Preda, Monica Irina Dutescu, Mihai Bojinca, Ioana Tieranu, Olivia Mihaela Popa

**Affiliations:** 1 Department of Gastroenterology, “Carol Davila” University of Medicine and Pharmacy, 020021 Bucharest, Romania; 2 Department of Gastroenterology, “Elias” Emergency University Hospital, 011461 Bucharest, Romania; 3 Molecular Biology Department, “Grigore Antipa” National Museum of Natural History, 011341 Bucharest, Romania; 4 Clinic of Internal Medicine II, Thüringen-Kliniken “Georgius Agricola”, 07318 Saalfeld, Germany; 5 “Prof. Dr. C. T. Nicolau” National Institute of Blood Transfusion, 011155 Bucharest, Romania; 6 Department of Rheumatology and Internal Medicine, “Carol Davila” University of Medicine and Pharmacy, 020021 Bucharest, Romania; 7 Department of Pediatrics, “Carol Davila” University of Medicine and Pharmacy, 020021 Bucharest, Romania; 8 Department of Immunology and Pathophysiology, “Carol Davila” University of Medicine and Pharmacy, 020021 Bucharest, Romania

The authors wish to correct the authorship of the original publication [1]. The corrected authorship is as follows: Andrei Ovidiu Olteanu followed by Elena Mirela Ionescu instead of the vice versa initial order. The authors apologize for any inconvenience caused and state that the scientific conclusions are unaffected. This correction was approved by the Academic Editor. The original publication has also been updated.
